# Identifying Distinct Implementation Patterns for Artifacts of Culture Change 2.0 Practices in Nursing Homes

**DOI:** 10.1093/geroni/igaf122.158

**Published:** 2025-12-31

**Authors:** Frances Yang, Clarissa Shaw, Carissa Coleman, Yelena Perkhounkova, Maria Hein, Amy Berkley, Maria Roche-Dean, Kristine Williams

**Affiliations:** University of Kansas, University of Kansas Medical Center, Kansas, United States; University of Iowa College of Nursing, Iowa City, Iowa, United States; University of Kansas Medical Center, Kansas City, Kansas, United States; University of Iowa, Iowa City, Iowa, United States; University of Iowa, Iowa City, Iowa, United States; University of Kansas School of Nursing, Kansas City, Kansas, United States; Kirkhof College of Nursing, Grand rapids, Michigan, United States; University of Kansas, Kansas City, Kansas, United States

## Abstract

The Artifacts of Culture Change 2.0 (ACC) is a self-assessment tool of 134 practices allowing nursing homes to evaluate and benchmark their culture related to person-centered care. Latent profile analysis was conducted in Mplus 8.11 to identify distinct patterns of culture change implementation across 147 nursing homes from 37 states. The analysis identified mean percent implementation ($\bar{\text{x}}$%i) across the five ACC practices groupings: 1) resident directed life, 2) being well known, 3) home environment and accommodation of needs and preferences, 4) family and community, and 5) leadership and team engagement. The overall implementation of culture change practices averaged 60.7% (SD + 15.7%, Range=19.8%-99.3%). Statistical criteria supported five implementation pattern profiles (Loglikelihood H0 -2925, AIC 5918, BIC 6020, SABIC 5913). The three most frequent patterns were categorized as ‘average implementers’ with medium implementation across all practices (n = 46, 31%), ‘low implementers’ with low scores across all practices (n = 43, 29%), and ‘team empowered’ with medium-high leadership and team engagement scores (n = 41, 28%). Two smaller groups emerged as ‘high implementers’ with high scores across all practices (n = 10, 7%) and ‘connection challenged’ that specifically struggled with family, community, leadership, and team engagement (n = 7, 5%). Resident’s being well known was the most highly implemented practice within each profile and family and community engagement was the lowest practice across the profiles. The next phase of this research will investigate whether these distinct implementation profiles are associated with resident and nursing home outcomes collected as part of our ongoing national trial on person-centered communication in nursing home care.

